# Better understanding of childhood asthma, towards primary prevention – are we there yet? Consideration of pertinent literature

**DOI:** 10.12688/f1000research.11601.1

**Published:** 2017-12-20

**Authors:** Michal Gur, Fahed Hakim, Lea Bentur

**Affiliations:** 1 Pediatric Pulmonary Institute and CF Center, Rappaport Children's Hospital, Rambam Health Care Campus, Haifa, Israel; 2 Rappaport Faculty of Medicine, Technion–Israel Institute of Technology, Haifa, Israel

**Keywords:** Asthma, wheezing, environmental, factors, prevention

## Abstract

Asthma is a chronic disease, characterized by reversible airway obstruction, airway inflammation and hyper-reactivity. The prevalence of asthma has risen dramatically over the past decade, affecting around 300,000,000 people. The etiology is multifactorial, with genetic, epigenetic, developmental and environmental factors playing a role. A complex interaction between the intrauterine environment, the developing immune system, the infant's microbiome and infectious organisms may lead to the development of allergic sensitization and asthma. Thus, a large number of studies have investigated the risk factors for childhood asthma, with a meticulous search of modifiable factors that could aid in primary prevention.

We present a current literature review from 2014-2017, as well as older classic publications, on the pathogenesis and the potential modifiable factors for primary prevention of asthma. No ideal preventive measure has yet been found. Rather, creating favorable prenatal and postnatal environments, minimal exposure to hostile environmental factors, prevention of infections in early life, allergic desensitization and nutritional modifications could possibly reduce asthma inception. In the era of personalized medicine, identifying individual risk factors and tailoring specific preventive measures is warranted.

## Introduction

Asthma is a chronic disease, characterized by episodes of reversible airflow obstruction. The prevalence of allergic diseases and asthma has risen substantially over the past decades. Currently, it is the most common non-communicable disease, affecting around 300,000,000 people, especially in developed countries, leading to enormous public health costs ^1^. In a recent study, the total annual health care expenditure attributable to asthma for school-aged children in the United States was 5.92 billion US dollars ^2^. Currently, no available therapeutic regimens can cure asthma, and the burden of asthma will continue to be driven by the increased but, as yet, poorly explained prevalence ^3^.

Population-based birth cohorts on asthma and allergies may provide insights into the development and natural history of the diseases. Over 130 birth cohorts have been initiated in the last 30 years ^4^. These birth cohorts have improved our understanding of asthma inception, progressions and persistency. Thus, they may help in targeting the ambitious and important goal of primary prevention of asthma. The Tucson birth cohort, developed in 1995 by Martinez *et al.* is the “classic” and most utilized model. This cohort proposed three categories of wheezing phenotypes at early age: transient wheezers (wheezing symptoms before 3 years, no wheezing at age 6), late onset wheezers (no wheeze until 3 years, wheezing at age 6), and persistent wheeze (wheezing in the first 3 years, wheezing at 6 years). This latter group of persistent wheezers can be divided into non-atopic and atopic. One third of all children aged 3 or younger had lower respiratory tract illnesses with wheezing; however, by the age of 6 close to 60 percent of these children no longer had wheezing symptoms. Transient early wheezers were found to have lower levels of lung function compared to other groups of wheezers, possibly reflecting congenitally smaller airways ^5^. Understanding the trajectory of early wheezing may help identify early predictors of later childhood persistent asthma. This is of utmost importance in identifying preventive interventions that could potentially reduce the inception of asthma ^6^.

The etiology of asthma is multifactorial; genetic, epigenetic, developmental and environmental factors play a role, as do the interactions between them ^1, 3^. Two important risk factors have been identified: the development of allergic sensitization and wheezing respiratory tract illnesses at an early age. Since both allergic sensitization and viral/bacterial illnesses occur in children who do not develop asthma, it is crucial to identify genetic and environmental factors that activate, interfere with and direct the immune system toward the development of asthma ^7^. Moreover, it has been found that environmental factors affecting a critical period in lung development (during pre- and postnatal periods) are associated with the development of allergic diseases and asthma. Epigenetic pathways could mediate the gene- environment interactions ^8^ and may explain the impact of external environmental factors on disease development.

Taking into account the significant burden of asthma, it is crucially important to find preventive measures. Strategies targeting asthma prevention can be primary (e.g. infants at high-risk for asthma) or secondary, dealing with children who have developed allergic sensitization or the first manifestations of allergic diseases (e.g. eczema or wheezing) ^9^. However, the heterogeneity and complex natural history of the disease serve as a barrier for the use of a single prevention strategy and suggest the importance of individualized risk assessment and multiple prevention measures with personalized primary prevention strategies ^3^. This review discusses research from the past 3 years, focusing on childhood asthma and primary preventions. We conducted a PubMed search of observational studies and clinical trials, including systematic reviews and meta-analyses, from 2014 to 2017. We also included older classic publications; thus, the search included studies dealing with potentially modifiable factors (both prenatal and postnatal) implicated in the inception of asthma. Environmental factors, early life respiratory infections, host factors and nutritional interventions will also be discussed.

## Environmental factors

### Favorable environment

***The “farm effect”.*** The importance of favorable environmental exposures in the development of asthma was demonstrated by epidemiologic studies showing significant protection from asthma and allergic diseases in children raised on traditional dairy farms. In particular, it was found that children who lived on farms were exposed to a greater variety of environmental microorganisms than controls. Endotoxin, a cell wall component of Gram negative bacteria, along with peptidoglycan, is widespread in stables and other farming environments and exposure to such microbial compounds was found to be inversely correlated with the risk of atopy ^10^. Higher diversity of microorganism exposure was correlated with a reduced prevalence of asthma and atopy ^11^. Compelling data exist showing that the “farm effect” ^12^ is not only present in childhood but also exerts an effect during pregnancy, influencing the developing fetal immune system. The farming environment has been linked with lower rates of asthma in offspring, although early-life farm exposure was shown to boost this effect ^13^. A recent study with the Amish community using traditional farming and the Hutterite community using industrialized farming evaluated asthma prevalence in children with similar genetic ancestries and lifestyles ^14^. Asthma prevalence and allergic sensitization was 4 and 6 times lower in the Amish community, whereas median endotoxin levels in Amish house dust was 6.8 times higher. The authors used a murine model of experimental allergic asthma to assess the effects of dust extracts found in Amish and Hutterite households on immune and airway responses. Experiments in humans and mice indicated that the traditional farm environment guards against asthma via interaction with and molding of the innate immune response. Hence, it is evident that altering innate immune signaling, as by farm environment, may be the primary target of asthma protection. However, the interventions required to create a farm effect without living on a farm are as yet unknown.

***Pets.*** Several prospective birth cohort studies found a decreased prevalence of atopic disease in children having daily contact with pets, in particular cats and dogs, during early infancy ^10^. Exposure to two or more dogs or cats in the first year of life was associated with a significantly lower risk of atopy (adjusted OR 0.23, 95% CI 0.09-0.60) ^15^. Another study found that living with a cat was inversely related both to having a positive skin test to cat (RR, 0.62 [0.47–0.83]) and incidence of physician-diagnosis of asthma (RR, 0.49 [0.28–0.83]). The effect was most pronounced among the children with a family history of asthma. Ownership of a dog resulted in weaker protective trends ^16^. A pooled analysis of individual participant data of 11 prospective European birth cohorts of 22,000 children concluded that pet ownership in early life did not appear to either increase or reduce the risk of asthma ^17^. However, a large nationwide cohort study (1,011,051 children) in Sweden found that dog exposure during the first year of life was associated with a decreased risk of asthma in school-aged children (OR 0.87, 95% CI 0.81-0.93) and in children 3 years or older (HR 0.90, 95% CI 0.83-0.99). Exposure to farm animals was correlated with a lowered risk of asthma in both children of school and preschool age. These results were independent of parental asthma ^18^.

However, the effect of exposure to dogs and farm animals was less pronounced in children younger than 3 years of age. These young children may reflect a group of children with transient wheeze and not persistent asthma ^5^. Taken together, most (but not all) studies support the theory of protective effects of having pets on asthma development.

***Day care attendance.*** The effect of day care attendance on asthma development is complex. In a study involving 1,035 children followed since birth as part of the Tucson study, children with older siblings or day care attendance were more likely to have frequent wheezing at the age of 2 years, but less likely to have frequent wheezing from the age of 6 to 13 years ^19^. Different results were obtained in a birth cohort of 762 children ^20^. In this study, day care attendance at 12 months was associated with an increased risk of asthma [OR 1.8, 95% CI 1.1-3.0]. A multivariate logistic model showed that day care attendance and number of lower respiratory infections at 12 months were associated with asthma [OR 1.2 (1.1-1.5); OR 1.4 (1.2-1.7), respectively]. Nevertheless, day care attendance of greater than 37.5 hours per week was associated with a lower risk of asthma [OR 0.6 (0.4-0.9)]. Hence, day care during infancy can either increase or reduce the risk of asthma.

### Prevention of allergic sensitization

There is a clear association between asthma and allergy ^21^. Most school age children and adolescents with asthma also have allergic sensitization. Early allergic sensitization is a risk factor for asthma development. In children of preschool age, researchers have found a sequential relationship of allergic sensitization followed by virus-induced wheezing. Exposure to allergens may increase asthma severity in susceptible patients (for example, an asthmatic patient exposed to dust mite allergens may develop increased airway hyper-reactivity) ^22^. Therefore, prevention of allergic sensitization is a major path for primary prevention of asthma.

A Cochrane review in 2009 found that multifaceted interventions (reducing exposure to both inhalant and food allergens) resulted in a significant decrease in asthma compared to usual care (<5 years: OR 0.72, 95% CI 0.54 to 0.96; >5 years: OR 0.52, 95% CI 0.32 to 0.85). Monofaceted interventions (reducing exposure to either inhalant or food allergens) did not produce significant effects ^23^. Another interventional study assessed the effectiveness of a multifaceted intervention program for the primary prevention of asthma in high-risk infants. The interventions were initiated shortly before birth and applied until 1 year of age, and included avoidance of house dust mites, pets, environmental tobacco smoke, encouragement of breast-feeding, and delayed introduction of solids. These measures resulted in a 4-fold reduction in the risk of asthma (adjusted OR, 0.26; 95% CI 0.08-0.88) in the children who did not develop atopy by 1 year of age; however, in children with early, persistent atopy, the risk of asthma was not reduced ^24^. Moreover, attempts to reduce exposure to house dust mites, known to be strongly associated with atopic sensitization and the development of asthma in childhood, were not found to be effective in the primary prevention of disease ^3^. Lynch *et al*. examined a birth cohort and a nested case-control study to assess the factors linking contact with allergens and bacteria in the first 3 years of life with the inception of recurrent wheeze and atopy. As the authors put forward, increasing allergen exposure in the first 3 years correlated with allergic sensitization, and allergic sensitization correlated with recurrent wheeze. However, there was a negative association between contact with cockroach, mouse and cat allergens in the first year and recurrent wheeze (OR 0.60, 0.65, and 0.75, respectively, *p*≤0.01) ^25^.

Overall, the reported nature of the relationship between exposure and sensitization varies widely among studies, from no significant association to a simple linear dose–response relationship or a ‘bell-shaped’ dose–response model with a protective effect of high allergen exposure. A recent study assessed the potential role of immunotherapy to alter the natural course of allergic march from allergic sensitization to asthma. The study included 812 children (5–12 years), with grass pollen allergic rhinoconjunctivitis and no medical history or signs of asthma. Children were double blinded and randomly allocated to receive immunotherapy or placebo for three years and were followed for two additional years ^26^. The study showed reduced risk of experiencing asthma symptoms and using asthma medication, but did not show an effect on the time to onset of asthma.

Hence, the relationship of atopic desensitization and asthma is complex and is likely determined by the type of allergen, the timing, pattern, route of exposure (inhaled, oral, transcutaneous), dose, as well as other environmental factors and individual genetic predispositions ^21^. Thus, the role and the measures of prevention of sensitization in the development of IgE-mediated sensitization asthma are yet to be determined.

### Hostile environment

***Air pollution.*** Air pollutants, representing a complex exposure to inorganic and organic components, are known to exacerbate asthma symptoms and might play a role in initiation of this disease. Particulate matter (PM) carries both environmental pollutants, such as polycyclic aromatic hydrocarbons (PAHs)—formed during incomplete combustion of fossil fuels and oil products—as well as agents causing immune stimulation—such as pollens, endotoxin and fungal spores. Air pollutants probably cause oxidative injury to the airways, leading to inflammation, remodeling, and an increased risk of sensitization. The idea that air pollution can cause exacerbations of pre-existing asthma is supported by evidence-based studies, but evidence suggests that air pollution might cause new-onset asthma as well, both in children and in adults ^27^. In a recent epidemiologic review, Schulz *et al*. concluded that early life and school-age exposures to air pollution has a negative impact on lung function, at least up to adolescence ^28^. In a Canadian birth cohort study, postnatal exposure to traffic-related air pollution increased the risk for the development of atopy to any allergens (adjusted OR 1.16; 95% CI 1.00-1.41) ^29^.

Gauderman *et al*. found an association between improvements in air quality in southern California and measurable improvements in lung-function development in children ^30^. Consequences of exposure to pollutants, pre- or postnatal, represent a complex interaction between the environment, the host and epigenetic factors. In children, PAH exposure has been associated with changes in DNA methylation, as well as impaired function of regulatory T cells ^31^. Epigenetic processes translate environmental exposures into regulation of the identity, gene expression profile, and activity of specific cell types that participate in the pathophysiology of the disease. Further discussion of the effects of air pollution is beyond the scope of this review.

***Tobacco smoke.*** There is evidence indicating a consistent detrimental effect of prenatal exposure and postnatal environmental smoking on childhood wheezing illnesses. Environmental tobacco smoke (ETS) during critical periods of lung development (prenatally, i.e. during pregnancy, and during early life) is considered a substantial risk factor for childhood allergic diseases. ETS induces over-expression of Toll-like receptors on the surface of the airway epithelium, increases oxidative stress and activates dendritic and innate lymphoid cells through the production of cytokines, such as IL-1, IL-25 and IL-33. The result is a higher susceptibility to allergen sensitization and a further risk for asthma ^32^. Genetic polymorphism on chromosome 17q21 was found to be predictive of childhood-onset asthma, and the risk was further increased by early-life exposure to environmental tobacco smoke ^33, 34^.

In a meta-analysis, ETS was associated with an increased risk of elevated specific IgE (OR 1.12; 95% CI 1.00-1.25) and positive skin prick test (OR 1.15; 95% CI 1.04-1.28). The relationship was stronger in young children and in prospective studies ^35^. In another meta-analysis of 79 prospective studies, ETS was found to increase the risk of wheeze by age 2 years by 70%; prenatal smoking was related to a 40% increased risk. The risk of asthma decreased with age (30% at age 3–4 years, 23% at 5–18 years) ^36^. In a cohort study of 27,993 mother–child pairs, children of mothers exposed to passive smoking whilst pregnant but no other smoking exposure had an increased tendency to develop wheeze up to 2 years old (OR 1.11; 95% CI 1.03–1.20) in comparison with control pairs. Exposure to passive smoke postnally, in addition to their mothers’ prenatal passive exposure, further increased the risk (OR 1.29; 95% CI 1.19–1.40). The risk was highest with passive neonatal exposure in addition to prenatal active smoking (OR 1.73; 95% CI 1.59–1.88) ^37^. Thus, ETS was found to be an important but avoidable risk factor for the development of allergic disease in children ^35^. Smoking is a modifiable risk factor in asthma. There is definitely a need for robust measures to reduce prenatal and postnatal smoking as a strategy for primary prevention of asthma.

## Early life respiratory infections

### Infections

Childhood asthma and infant respiratory viral infection are the most frequent chronic and acute illnesses of childhood, respectively. Over the years, it has been possible to make links between these diseases and spot common clinical traits. Early life respiratory syncytial virus (RSV) and human rhinovirus (HRV) lower respiratory tract infections (LRTIs) have been found to be strongly associated with increased asthma risk. During early infancy, RSV is a more common cause of severe LRTI. With advancing age, the situation is reversed, with HRV becoming more common. In spite of continuous research, the role of these respiratory viruses in the inception of asthma is still under debate ^38^.

***Respiratory syncytial virus (RSV).*** Severe RSV bronchiolitis requiring hospitalization is considered a risk factor for future asthma. In the RSV Bronchiolitis in Early Life Study, 50% of patients hospitalized with RSV had a physician diagnosis of asthma at age 7 years ^39^. Another study found that 21% of infants hospitalized for RSV bronchiolitis had asthma at age 6 years, compared to 5% in controls ^40^. There is a direct relationship between the severity of the initial RSV bronchiolitis and the risk of subsequent asthma, while environmental factors may further augment the risk ^41^. Thus, the association between early life RSV LTRI and later wheezing is consistent across most studies with large effect sizes and severity dose-response relationship.

The pathway from early-life infections with RSV to asthma is the result of complex interactions between the specific type of the virus, genetic, and environmental factors. RSV induces persistent airway damage and bronchial hyper-responsiveness ^42^. It has been suggested that RSV infection affects Th1/Th2 balance in early childhood, thereby inducing an atopic state and may, therefore, be involved in the inception of asthma ^43^. There is data that suggest that in utero exposure to RSV is followed by dysregulation of neurotrophic pathways, predisposing to postnatal airway hyperreactivity upon reinfection with the virus ^44^.

Palivizumab (monoclonal antibody) prophylaxis in the first year of life reduces recurrent wheezing in children aged 1 to 3 years ^40^. Palivizumab resulted in a relative reduction of 61% (95% CI 56-65) in the total number of wheezing days during the first year of life ^45^. However, the substantial cost, the need to treat a large population early in life (before RSV infection), and the need for parenteral administration limit its widespread use ^41^. While there is evidence that palivizumab prophylaxis reduces wheezing, its long-term impact on the development of atopic asthma remains controversial. In a follow-up study of children aged 2 to 5 years, palivizumab administration resulted in a 68% reduction in wheezing in the families of non-asthmatics and an 80% reduction in wheezing in non-atopic families, but no protection was achieved in atopic families ^46^. Similarly, in a recent prospective multicenter observational cohort study of 444 preterm infants, palivizumab prophylaxis administration significantly reduced subsequent physician diagnosis of recurrent wheezing up to 6 years, but did not reduce the incidence of atopic asthma, casting doubt on the suggestion that RSV prevention may decrease atopic asthma inception ^47^. Carroll *et al.* looked at infants at high-risk for severe RSV and whether a link could be found between better adherence to immunoprophylaxis and decreased childhood asthma. Analysis revealed that 70% or greater adherence decreased the odds of asthma compared to those with 20% or less adherence (OR 0.62; 95% CI 0.50-0.78) ^48^. Recently, in two prospective birth cohort studies, HRV infection was significantly more common among infants administered with RSV immunoprophylaxis (OR, 1.65; 95% CI 1.65-2.39) ^49^.

Taken together, the impact of palivizumab on the development of atopic asthma after RSV infection is still controversial. A definitive large-scale randomized clinical trial (RCT) measuring the effect of the prevention of RSV on childhood asthma is yet to be done. A large study evaluated 607 children aged 12 to 71 months at the beginning of a LRTI. Administration of azithromycin significantly reduced the odds of progression to severe LRTI (HR 0.64; 95% CI 0.41-0.98, p=0.04) ^50^. A relatively small double-blind placebo-controlled trial found a protective effect of azithromycin therapy during RSV bronchiolitis on subsequent recurrent wheeze ^51^. Currently, a larger study assessing asthma prevention following RSV by azithromycin is being conducted (NCT02911935).

Other strategies to prevent RSV are under investigation. There are some efforts to develop a specific vaccination against RSV. Recently, a genetically engineered, live attenuated vaccine was found to be effective in a phase I study ^52^. Another innovative approach includes immunization of pregnant women that potentially will decrease airway hyperreactivity upon postnatal reinfection with the virus ^53^.

In conclusion, although there is a clear association between RSV and asthma, the role of strategies to prevent RSV (palivizumab, azithromycin, vaccine) in asthma prevention has yet to be confirmed.

***Human rhinovirus (HRV).*** HRV has been increasingly recognized as an etiologic factor in preschool wheeze, as well as a significant risk factor in the development of asthma ^41^. Episodes of wheezing during which HRV was found in the upper airways have been found to be a strong predictor of subsequent asthma ^54^. HRV wheezing illnesses were found to increase the risk of asthma by 10-fold at 6 years of age ^55^. Lee *et al.* analyzed 1,445 samples collected from 209 infants enrolled in the COAST (Childhood Origins of ASThma) cohort study; these infants had an increased risk for developing allergies and asthma. HRV species A and C were about 7 times more likely to cause moderate to severe illness. These results firmly indicate that antiviral therapies aiming at lowering HRV-related morbidity in those infants at high risk ought to be directed towards HRV-A and HRV-C species.

As with RSV, the mechanism by which severe early-life HRV causes future asthma remains uncertain. Postulated mechanisms are airway epithelial injury and/or creating the appropriate pro-inflammatory allergenic milieu; alternatively, wheezing viral LRTI may serve as a marker for asthma susceptibility. Several studies suggest that the initial wheezing HRV LRTI may serve as a marker for asthma tendency, while early-life severe RSV bronchiolitis may have a causative role in the development of asthma ^41^. Thus, preventive measures are aimed at decreasing the incidence and severity of infections caused by HRV. A recent study ^56^ identified day-care attendance (OR 5.0; 95% CI 2.3-10.6), high eosinophil blood counts (OR 2.6; 95% CI 1.2-5.7) and exposure to tobacco smoke (OR 2.5; 95% CI 1.1-15.6) as significant risk factors for HRV LRTI. Hence, restricting children’s exposure to tobacco smoke may limit dissemination of viruses to younger children, counteract severe respiratory diseases, and thus may reduce sequelae.

An RCT assessed the efficacy of oral corticosteroid treatment during the first HRV LRTI to reduce the frequency of subsequent wheezing within 12 months. The study failed to show reduction in post HRV wheezing; however, children with a high viral load treated with prednisolone had a longer time to the next wheezing episode compared to placebo ^57^.

Strategies for developing an effective vaccine ^58^ or for preventing viral contact and invasion by forming a barrier on the host mucosa ^59^ are being developed; however, currently there is no approved strategy against HRV. Limiting viral spread is the main available protective measure.

## Host factors

### The microbiome

The whole host-microbe system benefits from the essential ecosystem services provided by microbial communities (microbiota) and the host environment in which they reside. Such services include making vital resources, nutrient bioconversion, and guarding against harmful microbes. Disease can arise from a shortfall in these beneficial functions or the maladaptive functions introduced by pathogenic microbes.

With the advent of 16s rRNA sequencing, a strong association between the host microbiome and asthma has emerged. The microbiome can be modulated by various environmental factors, including diet, prebiotic and probiotic, and early-life microbial exposures. Recently, the nasopharyngeal microbiome of 33 healthy infants was compared to 99 infants with confirmed RSV. The abundance of the dominant genera was significantly different between the groups, suggesting that RSV alters the infant nasopharyngeal microbiome and, thereby, may contribute to asthma development ^60^.

Affecting the microbial dysbiosis was suggested as a target for the prevention of asthma ^61^. Herein, we will discuss the potential role of a prebiotic and probiotic diet in the prenatal and postnatal period.

***Prenatal.*** Prenatal prebiotic and probiotic supplementation may alter maternal gut bacteria and influence maternal immune function. There is an inconsistent effect on the offspring of mothers supplemented during pregnancy, and a comprehensive 2016 World Allergy Organization review found a lack of evidence to support the use of prebiotics during pregnancy ^62^.

Maternal urinary tract infection during pregnancy increased the likelihood of asthma (OR 1.21; 95% CI 1.2-1.220). ^3^ Several studies have reported an association between the use of over-the-counter antipyretics during pregnancy or infancy and an increased risk (OR 1.26; 95% CI 1.02-1.58) of asthma in early childhood, but not mid-childhood ^63^. In a recent review, the maternal use of antibiotics or paracetamol during pregnancy was suggested as a modifiable risk factor for childhood asthma ^64^.

***Postnatal.*** Microbial gut colonization typically starts at the time of birth, and is influenced by the bacterial load of maternal microbiota, type of delivery (cesarean section vs. vaginal delivery), feeding (formula vs. breast-feeding), and the use of antibiotics.

In a retrospective cohort study including 321,287 births, there was no difference in the risk of asthma following planned compared to unscheduled cesarean surgery. However, compared to vaginal delivery, planned cesarean delivery resulted in a small increase in the risk of asthma requiring hospital admission (adjusted HR 1.22; 95% CI 1.11-1.34) and salbutamol inhaler prescription at age 5 years (adjusted HR 1.13; 95% CI 1.01-1.26) ^65^. In another study, cesarean section increased the risk of childhood asthma by 34% in univariate analysis and 11% after adjusting for other environmental exposures and covariates ^66^. Infants born via cesarean section have higher rates of *Clostridum*, *Klebsiella*, *Bacteroides*, and several other species at age 1 to 6 months, which predispose them to a higher risk for asthma, atopy and allergic rhinitis ^67^. In the newborn period, the gut microbiota plays a crucial role in maintaining the structure and function of the mucosal immune system. Gut-associated mucosal lymphoid tissue becomes reactive to pathogenic bacteria but tolerant to “beneficial” bacteria. T regulatory cells (Treg) are the key players in immunological tolerance; changes in their number or function are associated with the development of allergy ^67^. In a recent systematic review, the early microbiota of children who later developed allergies showed lower bacterial diversity; moreover, a predominance of Firmicutes, a higher prevalence of *Escherichia coli*, *Clostridium difficile*, and a lower prevalence of *Lactobacillus* were found ^68^.

In the Canadian Healthy Infant Longitudinal Development (CHILD) Study, Arrieta *et al.* evaluated the gut microbiota of 319 individuals. They found that those at risk of asthma showed transient gut microbial dysbiosis in their first 100 days of life, with reduced levels of *Faecalibacterium*, *Veillonella*, *Lachnospira*, and *Rothia*. Moreover, when these four bacterial taxa were introduced to germ-free mice, they showed decreased airway inflammation, suggesting a causal role of these bacteria in asthma development ^69^.

Several animal and human studies have examined the role of the postnatal use of prebiotics and probiotics in the prevention of allergic diseases. A meta-analysis reported data from 4,755 children (2,381 in the probiotic group, 2,374 controls). Infants treated with probiotics had a significantly lower risk of eczema (RR 0.78; 95% CI 0.69-0.89). No significant difference in terms of prevention of asthma (RR 0.99; 95% CI 0.77-1.27) or wheezing (RR 1.02; 95% CI 0.89-1.17) was found. Another meta-analysis concluded that controlled studies have not yielded sufficient evidence to date to recommend prebiotics and probiotics for the primary prevention of asthma ^70^.

Perinatal and postnatal suggested modifiable behaviors were natural childbirth, breastfeeding, increased outdoor activities, diet and the judicious use of antibiotics and antipyretics. These measures may help restore the neonatal microbiome and may reduce the risk for allergic diseases ^71^.

## Nutritional prevention

Dietary interventions during pregnancy are attractive because they are inexpensive and follow the accepted practice of folic acid supplementation ^3^. Several nutritional supplements have been investigated; we will discuss the main recent findings.

Current evidence suggests that maternal diet during pregnancy influences the developing immune system of the fetus. Interestingly, maternal weight gain or obesity during pregnancy was found not only to increase maternal asthma exacerbations ^72^ but also the risk for childhood asthma ^64^. Thus, efforts to prevent childhood asthma focus on early prenatal and postnatal interventions. The Mediterranean diet (MD) comprises fruit, vegetables and grains, a moderate intake of dairy products and a low intake of meat. This diet has been suggested to have a potential protective role in asthma. In a recent pilot study involving 30 pregnant women, the introduction of MD was feasible and acceptable, with 93% of participants retaining the diet ^73^. In a meta-analysis, MD was associated with a lower prevalence of “asthma ever” (OR -0.86; 95% CI 0.74–1.01), as well as “current wheeze” (OR -0.85; 95% CI 0.75–0.98) and “current severe wheeze” (OR -0.66; 95% CI 0.48–0.90) ^74^.

Thus, although MD has been suggested to be beneficial to general health, its impact on asthma prevention is still not proven and requires a large epidemiological study; assessing the mechanism, the relevant window of exposure and addressing specific components of the diet is warranted.

### Vitamins

Most studies focus on vitamin D supplementation and the results are inconclusive. Vitamin D has the ability to regulate inflammation and modulate immune responses and cell growth. Experimental data suggest that vitamin D may affect the developing lung and immune system during the prenatal and postnatal periods. Additionally, observational studies have suggested an association between maternal intake of vitamin D, cord blood vitamin D levels and persistent and recurrent wheezing in early childhood ^75^. In an RCT, 623 pregnant Danish women receiving 400 IU/d of vitamin D during the third trimester of pregnancy were randomized to receive an additional 2400 IU/d or placebo. Follow-up of the children (N=581) was completed when the youngest child reached age 3 years. Persistent wheeze was diagnosed in similar rates (16% and 20%) of children whose mothers received supplemental vitamin D and placebo, respectively (hazard ratio 0.76; 95% CI 0.52-1.12; p=0.16). The results suggest that 2800 IU/d during the third trimester of pregnancy cannot reduce the risk of persistent wheeze in the offspring through age 3 years. In the Vitamin D Antenatal Asthma Reduction Trial (VDAART) RCT, 881 pregnant women at risk of having children with asthma were randomized to 4,000 international units (IU)/d vitamin D or placebo plus 400 IU/d of vitamin D. Supplementation with 4400 IU/d resulted in a 20% reduction of recurrent wheeze or asthma (hazard ratio 0.8; 95% CI 0.6-1) that did not reach statistical significance (p=0.051) ^76^. A secondary analysis of the data revealed that the largest protective effect was found in women with higher initial vitamin D levels who were randomized to the intervention group (adjusted OR 0.13; 95% CI 0.02-0.99) ^77^. This suggests that higher vitamin D levels in early pregnancy may be required for asthma/recurrent wheeze prevention in early life.

A meta-analysis reporting data from 2,456 children demonstrated that prenatal supplementation of vitamin D significantly decreased recurrent wheeze (RR 0.812; 95% CI 0.67-0.98). Postnatal vitamin D administration was found to be associated with a reduction in upper respiratory tract infections ^78^; however, its role in primary asthma prevention remains unclear. Thus, a large randomized double-blind controlled study is currently being conducted to assess the role of postnatal vitamin D supplementations on multiple end points, including allergy, atopy and asthma later in life (NCT01723852) ^79^.

Vitamin C and E studies have not undergone meta-analysis due to high heterogeneity; these vitamins did not appear to have a significant preventive effect on recurrent wheeze ^80^.

To conclude, currently there is no sufficient evidence regarding any of the vitamins in the inception and prevention of asthma. Larger double-blind studies are required to recommend their routine use.

### Antioxidants and fish oil

Antioxidants reduce reactive oxygen species and there are several reports about the inverse associations between intake of antioxidants and allergic diseases. Total antioxidant capacity (TAC) assesses the combined activity of all the dietary antioxidants. A study in 2,359 Swedish children found that higher dietary TAC was inversely associated with the sensitization to aeroallergens (OR 0.73; 95% CI 0.55-0.97) and the risk of allergic asthma (OR 0.57; 95% CI 0.34-0.94). Interestingly, the relationship was modified by exposure to air pollution. A stronger inverse association between dietary TAC and allergy was observed in children with low exposure to air pollution. The authors postulated that high TAC may not be enough to counteract the high oxidative stress caused by air pollution ^81^.

More than a decade ago, Hodge *et al.* examined the relation between certain food consumption and asthma. They found a significantly reduced risk of current asthma in children who ate fresh, oily fish (OR 0.26; 95% CI 0.09-0.72; p<0.01) ^82^.

Later on, observational studies suggested an association between low levels of dietary fish-oil derived fatty acids (FA) and the risk of asthma and wheezing. In an RCT, 736 pregnant women were assigned to receive 2.4 gr of fish oil or placebo (olive oil). In the treatment group, there was a 30.7% reduction in the risk of persistent wheeze or asthma. The effect was most prominent in the women with low FA levels at randomization; moreover, the effect of the intervention remained at age 5 years, suggesting that it is not restricted to the “transient wheezers” ^83^.

Taken together, dietary interventions may aid in the primary prevention of asthma. Antioxidants, MD and fish oil seem to have a beneficial effect. Vitamin D is associated with asthma, but evidence for its role in primary prevention is still lacking. Other vitamins studied (such as C and E) failed to show a beneficial effect.

## Stress

Stress has been suggested to modify the normal lung morphogenesis and maturation during pregnancy and the postnatal period. Stress may affect neuroendocrine, autonomic and immune function programming, thereby leading to increased asthma inception. Prenatal maternal stress was found to alter innate and adaptive immune response in cord blood mononuclear cells, suggesting that prenatal stress may impact the expression of allergic diseases and increase the risk for later childhood wheezing ^84, 85^.

Postnatally, maternal behavior was found to influence the development of hypothalamic-pituitary-adrenal (HPA) responses to stress in rodents, mediated by changes in glucocorticoid receptor (GR) expression ^86^. Dreger *et al.* found that exposure to maternal distress restricted to the first year of life resulted in a 40% increase in cortisol levels in children; beyond the postnatal period, response to stress differed according to the presence of asthma ^87^. A recent study in a high-risk birth cohort found that maternal stress at age 2 and 3 years and maternal depression at any age were positively associated with recurrent wheeze (p<0.05 and p≤0.01, respectively) ^88^. Moreover, both active and passive stressors in asthmatic patients were associated with an increased activation of the sympathetic nervous system ^89^. Taken together, the data suggest that prenatal as well as postnatal maternal distress may contribute to asthma development in children; thus, lowering early life stress may help decrease asthma.

## Conclusions

Asthma is a chronic inflammatory disease, and genetic, infectious, nutritional and environmental factors play a role in its pathogenesis. In recent years, there has been some advance in the concept of primary prevention of asthma. However, there is no consensus on the relative importance of risk factors associated with asthma inception and none of the primary prevention or intervention strategies investigated has provided sufficient evidence to lead to widespread implementation in clinical practice. Current findings suggest that the major preventing measures during pregnancy are avoiding of passive and active smoking and the possible modification of maternal microbiome (e.g. lifestyle, diet, nutritional supplements). Avoiding unnecessary caesarean delivery is the main perinatal measure that may affect asthma. Postnatally, the most important measures are preventing severe neonatal respiratory infection, increasing favorable environment and behaviors (e.g. mimicking farm residence, breastfeeding) and decreasing hostile environments (e.g. smoking and air pollution). Unfortunately, gaps in knowledge still exist. The exact immune pathways that predispose certain infants (and not others) to asthma following early life viral infections are not fully understood. Additionally, although there is a clear association between allergy and asthma, its role in the primary prevention of asthma is under debate. Moreover, none of the suggested therapies or interventions can serve as a sole solution for the prevention of asthma inception. Instead, the combination of several pre- and postnatal factors, such as creating a favorable environment with minimal exposure to a hostile environment, attempts to beneficially affect the maternal and infant microbiome, with prevention of infections in early life, is expected to be more effective. This should be achieved by extensive educational and public health efforts to reduce tobacco smoking and air pollution, to implement dietary interventions in pregnant women, and to encourage breastfeeding and childhood vaccinations.

Future research should focus on the prevention of RSV and HRV, possibly by vaccine development. Moreover, in the era of personalized medicine, a test that would recognize the specific asthma phenotype and endotype of each patient, as well as his or her own risk factors, would enable tailoring specific preventive measures for the individual patient.
